# Objects and List of Meetings

**Published:** 1887-06-04

**Authors:** 


### THE HOSPITALS WEEK.

*From Monday, June 13th, to Saturday, June 18th, 1887.*

ITS OBJECTS ARE :—To extend the interest and support already given to the Hospitals and Medical Charities, by arousing and fixing public attention to the following facts :

(1) The pressing pecuniary necessities of these institutions ; and

(2) The importance of their efficient maintenance to all classes of the community, from the highest to the lowest.

At the meetings, which will present many features of special interest, it is not proposed to make any collection, but to invite all intelligent citizens of every class to make a point of—

(*a*) Attending some place of worship on Hospital Sunday, June 27th, 1886 ;

(*b*) Sending a contribution direct to the Lord Mayor at the Mansion House, or

(*c*) Placing a donation in the boxes which will be fixed outside the Mansion House for the convenience of those who prefer to give in that way.

### LIST OF THE MEETINGS

| PROPOSED PLACES AND DAYS OF MEETINGS. | CHAIRMEN AND SPEAKERS. |
|---|---|
| 1.—Newington Parish Mission Hall, Kennington Road, S.E., MONDAY, 13th June, 8.30 p.m. | Sir Wm. Plowden, M.P., etc.<br>Rev. G. T. Palmer.<br>Rev. C. H. Spurgeon.<br>Sir Andrew Clark, Bart., M.D., etc.<br>Hon. Conrad Dillon.<br>Sir Sydney H. Waterlow, Bart. |
| 2.—Mansion House, E.C., TUESDAY, 14th June, 3 p.m. | The Rt. Hon. the Lord Mayor.<br>Marquess of Salisbury.<br>The Rt. Rev. Bishop of London.<br>Rev. H. Allon, D.D.<br>Henry Irving, Esq. |
| 3.—St. Andrew's Hall, Newman Street, W., WEDNESDAY, 15th June, 8 p.m. | Lord Charles Beresford, M.P., etc.<br>Colonel Duncan, M.P.<br>H. L. W. Lawson, Esq., M.P.<br>Major Ross, M.P.<br>Arthur Baumann, Esq., M.P. |
| 4.—Kensington Town Hall, THURSDAY, 16th June, 4 p.m. | Duke of Argyll, K.G., etc.<br>Earl Cadogan.<br>Sir A. Borthwick, M.P.<br>Hon. and Rev. E. Carr-Glyn.<br>The Cardinal Manning. |
| 5.—Wellington Hall, Islington, THURSDAY, 16th June, 8 p.m. | Rev. H. W. Barlow, B.D.<br>Sir A. K Rollit, M.P., etc.<br>Cowley Lambert, Esq., M.P.<br>R. Chamberlain, Esq., M.P.<br>G. C. T. Bartley, Esq., M.P. |
| 6.—Westminster Town Hall, FRIDAY, 17th June, 4 p.m. | Duke of Westminster, K.G., etc.<br>Very Rev. Dean of Westminster.<br>The Cardinal Manning.<br>Lord Randolph Churchill.<br>A. B. Burdett-Coutts, Esq., M.P., etc. |
| 7.—Stratford Town Hall, FRIDAY, 17th June, 8 p.m. | John Meeson, Esq., Mayor of Stratford<br>Earl of Rosebery.<br>Sir Edm. Hay Currie.<br>J. F. Fulton, Esq., M.P.<br>E. Rider Cook, Esq. |
| 8.—The People's Palace, Mile End Road, SATURDAY, 18th June, 3 p.m. | H.R.H. the Duke of Cambridge, K G.<br>Sir Edm. Hay Currie.<br>Right Hon. C. T. Ritchie, M.P., etc.<br>Right Rev. Bishop of Bedford.<br>Rev. G. S. Reaney. |

THE FOLLOWING MEETINGS WILL PRECEDE AND BE INTRODUCTORY TO "THE HOSPITALS WEEK."

| PROPOSED PLACES AND DAYS OF MEETINGS. | CHAIRMEN AND SPEAKERS. |
|---|---|
| Lambeth Palace Library, MONDAY, 6th June, 3.30 p.m. | Archbishop of Canterbury.<br>Viscount Lewisham, M.P.<br>Rev. the Hon. Canon Pelham.<br>Rev. Canon Erskine Clarke.<br>General Fraser.<br>Sir W. McArthur, K.C.M.G.<br>W. Gent-Davis, Esq., M.P. |
| Paddington Vestry Hall, TUESDAY, 7th June, 8 p.m. | Baron F. de Rothschild.<br>Rev. Walter Abbott, M.A.<br>Sir John Kennaway, M.P.<br>Hon. Henry Noel, J.P.<br>E. Boulnois, Esq., J.P.<br>Henry C. Burdett, Esq.<br>E. Parker Young, Esq. |
| Blackheath Lecture Hall, WEDNESDAY, 8th June, 8 p.m. | Baron de Worms, M.P.<br>H. Fell Pease, Esq., M.P.<br>W. J. Evelyn, Esq., M.P. |
| Hampstead Vestry Hall, WEDNESDAY, 8th June, 8 p.m. | Lord William Compton (Chair).<br>Walter Besant, Esq.<br>Lord Aberdare.<br>Sir Joseph Fayrer.<br>Sir S. H. Waterlow, Bart.<br>Rev. John Kennedy, D.D. |
| Shoreditch Town Hall, SATURDAY, 11th June, 3 p.m. | Right Rev. Bishop of Bedford.<br>Rev. Septimus Bass, LL.B.<br>Sir Sydney H. Waterlow, Bart.<br>The Marquess of Lorne.<br>Sir Charles Russell, Q.C., M.P.<br>C. Bigwood, Esq., M.P.<br>Rev. W. Cuff. |

*** The meetings will all be free and open, and no tickets of admission will be required.

